# High-Fat Diet Alleviates Neuroinflammation and Metabolic Disorders of APP/PS1 Mice and the Intervention With Chinese Medicine

**DOI:** 10.3389/fnagi.2021.658376

**Published:** 2021-06-08

**Authors:** Xiaorui Fan, Bin Liu, Junyi Zhou, Xinru Gu, Yanyan Zhou, Yifei Yang, Feifei Guo, Xiaolu Wei, Hongjie Wang, Nan Si, Jian Yang, Baolin Bian, Haiyu Zhao

**Affiliations:** ^1^School of Chinese Pharmacy, Beijing University of Chinese Medicine, Beijing, China; ^2^Institute of Chinese Materia Medica, China Academy of Chinese Medical Sciences, Beijing, China

**Keywords:** Alzheimer's disease, high fat diet, neuroinflammation, metabolism, Huanglian Jiedu decoction, gut microbiota

## Abstract

Alzheimer's disease (AD) is a progressive neurodegenerative disease caused by the complex interaction of multiple mechanisms. Recent studies examining the effect of high-fat diet (HFD) on the AD phenotype have demonstrated a significant influence on both inflammation and cognition. However, different studies on the effect of high-fat diet on AD pathology have reported conflicting conclusions. To explore the involvement of HFD in AD, we investigated phenotypic and metabolic changes in an AD mouse model in response to HFD. The results indicated there was no significant effect on Aβ levels or contextual memory due to HFD treatment. Of note, HFD did moderate neuroinflammation, despite spurring inflammation and increasing cholesterol levels in the periphery. In addition, diet affected gut microbiota symbiosis, altering the production of bacterial metabolites. HFD created a favorable microenvironment for bile acid alteration and arachidonic acid metabolism in APP/PS1 mice, which may be related to the observed improvement in LXR/PPAR expression. Our previous research demonstrated that Huanglian Jiedu decoction (HLJDD) significantly ameliorated impaired learning and memory. Furthermore, HLJDD may globally suppress inflammation and lipid accumulation to relieve cognitive impairment after HFD intervention. It was difficult to define the effect of HFD on AD progression because the results were influenced by confounding factors and biases. Although there was still obvious damage in AD mice treated with HFD, there was no deterioration and there was even a slight remission of neuroinflammation. Moreover, HLJDD represents a potential AD drug based on its anti-inflammatory and lipid-lowering effects.

## Introduction

Alzheimer's disease (AD) is a neurodegenerative disorder characterized by the pathological hallmarks of progressive cognitive decline, extracellular accumulation of amyloid-β (Aβ) plaques and intracellular accumulation of neurofibrillary tangles. Diet and nutrition display potential for non-pharmacological AD prevention (Hill et al., 2019); however, different studies examining the effect of a high-fat diet (HFD) on AD pathology in AD models have reported conflicting conclusions. Ordinarily, systemic inflammation and obesity caused by HFD are likely to interfere with immunological processes of the brain and further promote disease progression (Heneka et al., 2015; Nam et al., 2017). And study showed that the adversely affect can be reversed by low-fat diet, at least partially, even after 7 months of HFD feeding when neuropathological burden was reasonably advanced (Walker et al., 2017). However, other studies found that HFD have no effect on Aβ or tau (Studzinski et al., 2009; Herculano et al., 2013; Knight et al., 2014) or cognitive performance (Elhaik Goldman et al., 2018). In all the related research, potential genetic variability in different mouse strains and the age-related factors may explain the high variability of the results. In APP knock-in mouse model, carrying a humanized Aβ sequence with the Swedish mutation (KM670/671NL), this paper shown that high fat diet treatment, despite inducing obesity and a T2DM peripheral phenotype, failed to trigger AD pathology (Salas et al., 2018). HFD treated Tg2576 mouse had no effect on the level of amyloid beta_1−42_ in the cortex and shown more anxious, but had better learning which related to the improvements of BBB function (Elhaik Goldman et al., 2018). HFD had only a limited effect upon learning and memory in C57BL/6 mice despite broad peripheral changes (Mielke et al., 2006; Kesby et al., 2015). These data therefore suggest that different modes of memory are differentially sensitive to the effects of a high-fat diet. Research on the longitudinally the effect of a high-fat diet in both control non-Tg and 3xTgAD mice found that effects on memory were transient as at the age of 11–12 and 15–16 months no difference in time spent in the target quadrant was observed between 3xTgAD or non-Tg mice on either a control or high-fat diet (Knight et al., 2014).

A number of factors have been proposed to cause HFD-induced damage to the brain, especially in response to aging, including oxidative stress, insulin resistance, inflammation, and changes in vascularization/BBB integrity (Freeman et al., 2014). Therefore, it is necessary to explore the specific effect of HFD on metabolism in AD. HFD-related obesity has been reported by many studies to be involved in the regulation of gut microbiota, cholesterol circulation, and liver damage (DeAngelis et al., 2005; Kim et al., 2015). Because total serum cholesterol levels represent the most general change among the effects of high-fat diet (Guay et al., 2012), cholesterol that is excreted with bile acids (BAs) is a relevant host factor that modulates the gut microbiota (Martinez et al., 2013). Therefore, we hypothesized that altered gut microbiota was the more important metabolic change in response to HFD intervention. Emerging evidence from recent studies indicates that neurodegenerative diseases might be directly associated with the impairment of gut microbial flora (Li B. et al., 2019). Disturbances in the microbiota influence the formation of the amyloid beta peptide and exacerbate neurodegeneration through the upregulation of neuroinflammatory processes (De-Paula et al., 2018). The “gut-brain metabolic axis” facilitates bidirectional chemical communication between the central and enteric nervous systems (Nicholson et al., 2012; Wang and Wang, 2016). This metabolic axis is thought to be involved in the regulation of multiple host metabolic pathways in which the levels of short-chain fatty acids, hormones, neurotransmitters, amines, lipid metabolites, and others are regulated by gut microbiome activity (Nicolas and Chang, 2019).

The goal of the present study was to characterize diet-related effect on AD models and the therapeutical effects of HLJDD. In the present study, we performed a detailed examination of the effects of HFD on AD pathological characteristics, including the cognitive capacity, pathological slices and the neuroinflammation. Neurotransmitters, PUFAs and their derivatives in the central nervous system (CNS), BAs in the periphery were systematically detected by the UPLC-MS/MS to identify the general metabolic dysfunction, which have been as factors that may lead to impaired cognitive function. In addition, gut microbiota was analyzed to reveal the vital function of the gut-brain metabolic axis in HFD treated AD mice.

## Materials and Methods

### Animals

Four-month-old SPF grade male C57BL/6J-TgN (APP/PS1) transgenic mice and C57BL/6J wild type mice (Beijing HFK Bioscience CO., LTD, Production license number SCXK 2014-004) were used in this study. All experiments and animal care in this study were conducted in accordance with the National Institutes of Health Guide for the Care and Use of Laboratory Animals (NIH Publications No. 8023, revised 1978) and the Provision and General Recommendation of Chinese Experimental Animals Administration Legislation. The study was approved by the Institutional Animal Care and Use Committee of the Beijing animal science CO., LTD and the animal ethics approval number was IACUC-2018100605. Animals were housed in a single cage with chow and water *ad libitum* and a 12 h light-dark cycle and kept under a consistent temperature of 21°C. Wild type mice were assigned to the normal group and fed a normal chow diet. APP/PS1 mice were randomly allocated into one of 6 groups: one was fed a normal chow diet (the AD group), and the others were fed a HFD containing 63.6% basic feed, 15% lard, 20% sucrose, 1.2% cholesterol, and 0.2% cholate (Beijing Keao Xieli Feed CO., LTD). One of the HFD experimental groups was the AD + HFD group, and the other four were HLJDD-L, HLJDD-H, berberine (Ber), and donepezil (Don) groups. The Huanglian Jiedu decoction was prepared in our laboratory as previously described (Yang et al., 2013). Our research was a preventive protocol, and the gavage doses were 172 mg/kg/d (HLJDD-L) and 344 mg/kg/d (HLJDD-H) for 6 mouths. Oral gavage of 50 mg/kg/d berberine (H51022193) and 2 mg/kg/d donepezil (1705051) once daily was used as a positive control. Animal weights were recorded every 3 weeks.

### Morris Water Maze Test

The MWM test was performed to assess spatial memory. In acquisition trial, gently place the mouse into the water, facing the edge of the pool. If the mouse finds the platform before the 90 s cut-off, allow the mouse to stay on the platform for 10 s then return it to its home cage. Otherwise, place the mouse on the platform and allow it to stay there for 20 s. The mouse was trained in different direction. Repeat for all mice in the trail in the next 3 days. In probe trial, remove the platform from the pool and the test time was 60 s. Escape latencies, time spent or distance traveled in the target quadrant and platform-crossing times were recorded and analyzed using the analysis management system (Beijing Zhongshi Kechuang CO., LTD).

### Tissue Collection

After the MWM test, all animals had a 2-day rest, and then mice were deeply anesthetized via an intraperitoneal injection of sodium pentobarbital for sampling of heart blood. Brains were removed and immediately frozen in dry ice prior to storage at −80°C for subsequent metabolic analyses. For immunohistochemical analyses, removed brains were postfixed in 4% paraformaldehyde (PFA) solution and then immersed in a 4% PFA solution. Feces were collected before the day animals were euthanized, and the intestinal contents were also collected when animals were dissected and frozen at −80°C.

### Immunohistochemistry

Coordinates of the targets in mouse brains were determined using The Rat Brain in Stereotaxic Coordinates of Paxinos and Watson (fifth edition, page 53–63). Three-millimeter-thick brain tissues were dehydrated using gradient alcohol solutions, embedded in paraffin and cut into 4-μm sections, and dehydrated. Thin sections were dewaxed and rehydrated in distilled water for hematoxylin and eosin, Congo red and immunohistochemistry staining.

We performed antigen retrieval with citrate buffer (pH 6.0) treatment, and sections were blocked with endogenous peroxidase and washed in phosphate buffered saline (PBS) (pH 7.4) 3 times. Then, sections were blocked in 5% normal goat serum and 1% bovine serum albumin (BSA) with 0.3% Triton-X 100 in PBS for 30 min at room temperature. Sequential incubation with the primary antibodies, rabbit anti-beta amyloid 1–42 (1:1000, 1% BSA, 0.3% Triton-X100/PBS, ab201060), rabbit anti-GFAP (1:1000, 1% BSA, 0.3% Triton-X100/PBS, ab7260) and rabbit anti-MHC class II antibody (1:200, 1% BSA, 0.3% Triton-X100/PBS, ab180779), was performed overnight at 4°C. After washing in PBS five times, sections were sequentially incubated with HRP anti-rabbit IgG (peroxidase) polymer (PV-9001, Beijing Zhongshan Golden Bridge Biotech, China) for 20 min. Color development was performed by adding freshly prepared diaminobenzidine (DAB) for 30 s. Sections were counterstained in hematoxylin for 5 min, dehydrated, cleared and sealed with neutral gum. A slice scanner (Aperio, American) was used for recording and analysis.

### Biochemical Parameters

Brain tissue was homogenized in liquid nitrogen and subpacked. Then, NP-40 protein lysis buffer was added. After centrifugation, the supernatant was removed. Inflammatory factors, including TNF-α, IL-1β, and IL-6, were detected using ELISA kits (VAL609, VAL601, and VAL604).

Meso scale discovery (MSD) multispot assay system proinflammatory panel 1 (mouse) kits (K15048D) were used to measure ten inflammatory cytokines (IFN-γ, IL-1β, IL-2, IL-4, IL-5, IL-6, KC/GRO, IL-10, IL-12p70, and TNF-α) in serum. Total serum levels of cholesterol (TC), triglycerides (TG), high-density lipoprotein cholesterol (HDL-C), low-density lipoprotein cholesterol (LDL-C), free fatty acids (FFAs), albumin, and C-reactive protein (CRP) were determined using an automatic biochemical analyzer (AU480, Beckman Coulter, Inc., Brea, CA, USA).

### RT-PCR

Total RNA was extracted from brain tissue using TRIzol reagent (ELK Biotechnology, China) according to the manufacturer's instructions. RNA concentrations were equalized and converted to cDNA using the EntiLink™ 1st Strand cDNA Synthesis Kit (ELK Biotechnology, China). Gene expression was measured using a StepOne™ Real-Time PCR system. The sequences of primers used in these experiments are listed in the Supplementary Material.

### Western Blot Analysis

Brain tissues were lysed in precooled RIPA buffer with the protease inhibitor PMSF (Amresco), and protein concentrations were determined using a BCA protein assay kit. Protein samples were separated on 12% sodium dodecyl sulfate polyacrylamide gels electrophoresis (SDS-PAGE) and transferred onto NC membranes. Then, membranes were blocked in 5% non-fat milk for 30 min at room temperature and incubated with primary antibodies (rabbit polyclonal NR1H3 antibody, 1:1000; rabbit polyclonal PPARγ antibody, 1:4000; mouse monoclonal APOE antibody, 1:5000; rabbit polyclonal 5 lipoxygenase antibody, 1:200; rabbit polyclonal COX2 antibody, 1:1000) overnight at 4°C. Membranes were then washed and incubated with HRP-conjugated goat anti-rabbit and HRP-conjugated goat anti-mouse (1:10,000) secondary antibodies for 40 min at room temperature followed by development using ECL detection. The obtained bands were then scanned and analyzed using ImageJ software, and band density (IOD) was assessed using Total Lab Quant V11.5 (Newcastle upon Tyne, UK).

### Metabolic Phenotyping

#### Neurotransmitters

Brain tissue (50 mg) was weighed, and 1 mL of 80% acetonitrile solution (containing 0.2% formic acid) was added and centrifuged at 3,000 rpm, 0°C for 60 s, and ultrasonication was performed for 60 s. Samples were centrifuged again at 12,000 rpm, 0°C for 15 min. Then, 10 μL of 1% ascorbic acid, 10 μL of internal standard solution and 380 μL of methanol (with 0.2% formic acid) were added to 100 μL of supernatant, which was vortexed for 180 s and centrifuged at 12,000 rpm at 0°C for 15 min. After centrifugation, the supernatant was used for UPLC–MS/MS analysis. Each standard was precisely weighed, and 50% methanol solution was added to prepare the 2 mg/mL mixed standard stock solution. According to the abundance of each substance in the sample, each stock solution was precisely added at a corresponding volume into the mixed standard. The standard was diluted with 50% methanol, and the standard synephrine was accurately weighed and prepared in a 10 μg/mL internal standard solution. All solutions were stored at −80°C.

Gradient chromatographic separation was performed on a Waters PFP C18 column (2.1 × 100 mm, 1.7 μm). Mobile phase A consisted of 0.05% formic acid in acetonitrile. Mobile phase B consisted of 0.05% formic acid in water. Gradient elution was as follows: 0–4 min, 2% A; 4–6 min, 2–95% A; and 6–10 min, 95–95% A. The column temperature was 40°C, the flow rate was 0.3 mL/min, and the injection volume was 5 μL. Mass spectrometry analysis was performed using an electrospray ion source (ESI), and detection was performed in the multireaction ion monitoring (MRM) positive mode. The temperature was 550°C, the curtain gas (CUR) pressure was 35 V, the atomization gas pressure (GS1) was 55 psi, and the auxiliary gas flow rate (GS2) was 55 psi. The MRM ion pair transitions and collision energy levels of each component are listed in the Supplementary Material.

#### Polyunsaturated Fatty Acids and Metabolites

Sample preparation referenced a previous report (Yue et al., 2007). Briefly, 1 ng of 20-HETE-d6, 1 ng PGE_2_-d5 and 100 ng AA-d8 internal standard solutions were added. After vortex mixing, nitrogen blowing was performed by heating in 37°C water. Finally, the sample was redissolved in 50 μL of methanol and analyzed by LC-MS/MS. Standards of each polyunsaturated fatty acid and their metabolites were accurately weighed and diluted with methanol solution to prepare the mixed lipid peroxide standard stock solution of 200 ng/mL and PUFA standard stock solution of 200 μg/mL. Standards of 20-HETE-d6, PGE_2_-d5, and AA-d8 were weighed and prepared in 500, 500 ng/mL and 5 μg/mL internal standard stock solutions. All solutions were stored at −80°C.

Gradient chromatographic separation was performed on a Waters BEH C_18_ column (2.1 × 100 mm, 1.7 μm). Mobile phase A consisted of 0.05% formic acid in acetonitrile. Mobile phase B consisted of 0.05% formic acid in water. Gradient elution was as follows: 0–8 min, 60% A; 8–8.5 min, 60–75% A; and 8.5–11.5 min, 75–100% A. The column temperature was 40°C, the flow rate was 0.25 mL/min, and the injection volume was 5 μL. Mass spectrometry analysis was performed using ESI. Detection was performed in MRM negative mode. The temperature was 550°C, CUR pressure was 35 V, GS1 was 55 psi, and GS2 was 55 psi. MRM ion pair transitions and collision energy levels of each PUFA and its metabolites are listed in the Supplementary Material.

#### Bile Acids

Serum samples were melted at 4°C, and simple protein precipitation using methanol was used as follows: 130 μL of methanol was added to 50 μL serum samples, spiked with 20 μL of IS, and the sample was vortexed for 20 min and centrifuged at 12,000 rpm and 4°C for 5 min. The supernatant was collected, and 5 μL was injected into the LC–MS/MS system for analysis. Bile acid standard stock solutions were prepared by dissolving the respective 17 BAs in the appropriate amount of methanol to obtain mixed stock solutions of 1 mg/mL. Then, the solution was further diluted with methanol:water (3:1) to obtain a series of standard stock solutions. CA-d4 and LCA-d4 mixed solutions were prepared in methanol and further diluted with methanol to obtain a 2 μg/mL internal standard solution.

Chromatographic separation was performed on an ACQUITY HSS T3 column (2.1 × 100 mm, 1.8 μm). The mobile phase consisted of H_2_O:acetonitrile (10:1, containing 1 mmol/L ammonium acetate) (A) and acetonitrile:isopropanol (1:1) (B) at a total flow rate of 0.26 mL/min. The gradient profile for the LC pumps under the chromatography conditions were as follows: 0–1 min, 100% A; 1–2 min, 100–60% A; 2–6 min, 60–50% A; 6–8 min, 50–30% A; 8–9 min, 30–0% A; 9–12 min, 0%; 12–12.1 min, 0–100% A; and 12.1–13 min, 100% A. The injection volume of all samples was 5 μl. The column temperature was set at 50°C. For MS detection, the ESI source was operated in the negative ion mode to produce MS/MS spectra. The parameters were as follows: capillary 2 kV; cone 21 V; source temperature 150°C; desolvation temperature 600°C; cone gas flow 150 L/Hr; and desolvation gas flow 1,000 L/Hr. The MRM ion pair transitions and collision energy levels of each component are listed in the Supplementary Material.

#### Short-Chain Fatty Acids

Fecal short-chain fatty acids (SCFAs) were also analyzed. Briefly, 1,000 μL of ethanol (containing 0.5% hydrochloric acid, *v/v*) and 10 μL of internal standard (acetic acid-d4) were added to 25 mg of each sample. The mixture was vortexed and ultrasonicated for 40 min and centrifuged at 14,000 rpm for 10 min. Supernatants were analyzed by Agilent 7890-5977 gas chromatography–mass spectrometry (Agilent Technologies, Santa Clara, CA, USA).

The chromatographic column was a DB-FFAP (30 m × 0.25 mm i.d., 0.25 μm film, Agilent Technologies). One microliter of sample was automatically injected in the splitless mode, which was maintained at 250°C. Helium was used as a carrier gas at a flow rate of 1 mL/min in constant flow mode. The oven program was set at an initial temperature of 50°C for 2 min and was then increased to 120°C at a rate of 15°C/min, to 170°C at a rate of 5°C/min, and was finally increased to 240°C at a rate of 15°C/min and held at 240°C for 3 min. The electron impact ionization was 70 eV, and data were acquired in full scan mode and SIM mode. Identification of each SCFA was confirmed by comparing the mass spectra and retention times. Acquired tandem mass spectra were matched against the NIST Standard reference database 1A. The quantifiers for acetic acid, propionic acid, butyric acid, isobutyric acid, valeric acid, isovaleric acid and acetate d3 (ISTD) were m/z 43, 74, 43, 60, 60, 60, 60, and 46.

#### Microbial Diversity Analysis

Intestinal content was collected when animals were dissected and was stored at −80°C. DNA extraction and PCR amplification were subsequently performed. Microbial DNA was extracted from intestinal content samples using the E.Z.N.A.^®^ soil DNA Kit (Omega Biotek, Norcross, GA, U.S.) according to the manufacturer's protocols. The final DNA concentration and purification were determined by a NanoDrop 2000 UV-vis spectrophotometer (Thermo Scientific, Wilmington, USA), and DNA quality was checked by 1% agarose gel electrophoresis. The V3–V4 hypervariable regions of the bacterial 16S rRNA gene were amplified with the primers 338F (5′- ACTCCTACGGGAGGCAGCAG-3′) and 806R (5′-GGACTACHVGGGTWTCTAAT-3′) by a thermocycler PCR system (GeneAmp 9700, ABI, USA). PCRs were performed in triplicate in a 20 μL mixture containing 4 μL of 5 × FastPfu Buffer, 2 μL of 2.5 mM dNTPs, 0.8 μL of each primer (5 μM), 0.4 μL of FastPfu Polymerase, and 10 ng of template DNA. The resulting PCR products were extracted from a 2% agarose gel and further purified using the AxyPrep DNA Gel Extraction Kit (Axygen Biosciences, Union City, CA, USA) and quantified using QuantiFluor™-ST (Promega, USA) according to the manufacturer's protocol.

Illumina MiSeq sequencing was performed using purified amplicons, which were pooled in equimolar amounts and subjected to paired-end sequencing (2 × 300) on an Illumina MiSeq platform (Illumina, San Diego, USA) according to the standard protocols by Majorbio Bio-Pharm Technology Co., Ltd. (Shanghai, China). Processing of sequencing data involved using raw fastq files, which were demultiplexed, quality-filtered by Trimmomatic and merged by FLASH with the following criteria: (i) reads were truncated at any site receiving an average quality score <20 over a 50-bp sliding window; (ii) primers were exactly matched, allowing 2 nucleotide mismatches, and reads containing ambiguous bases were removed; and (iii) sequences whose overlap was longer than 10 bp were merged according to their overlapping sequence. Operational taxonomic units (OTUs) were clustered with a 97% similarity cutoff using UPARSE (version 7.1 http://drive5.com/uparse/), and chimeric sequences were identified and removed using UCHIME. The taxonomy of each 16S rRNA gene sequence was analyzed by the RDP Classifier algorithm (http://rdp.cme.msu.edu/) against the Silva (SSU123) 16S rRNA database using a confidence threshold of 70%.

### Statistical Analysis

For all experiments, at least three replicates were analyzed. All quantitative data are expressed as the mean ± standard error of the mean (SEM) and were analyzed using unpaired Student's *t*-test for two groups and one-way ANOVA for five groups, as indicated, followed by the LSD and Games-Howell post-test. Statistical calculations were performed using IBM SPSS Statistics software. Differences with statistical significance are denoted by *p* ≤ 0.05. Histograms were drawn using GraphPad Prism (San Diego, CA).

### Data Availability

The datasets generated and analyzed during the current study are available from the corresponding author upon reasonable request.

## Results

### Pathology and Biochemical Parameters in APP/PS1 Mice

#### HFD had no Effect on Cognitive Impairment or Cerebral Pathology in APP/PS1 Mice

**Figure 1:**
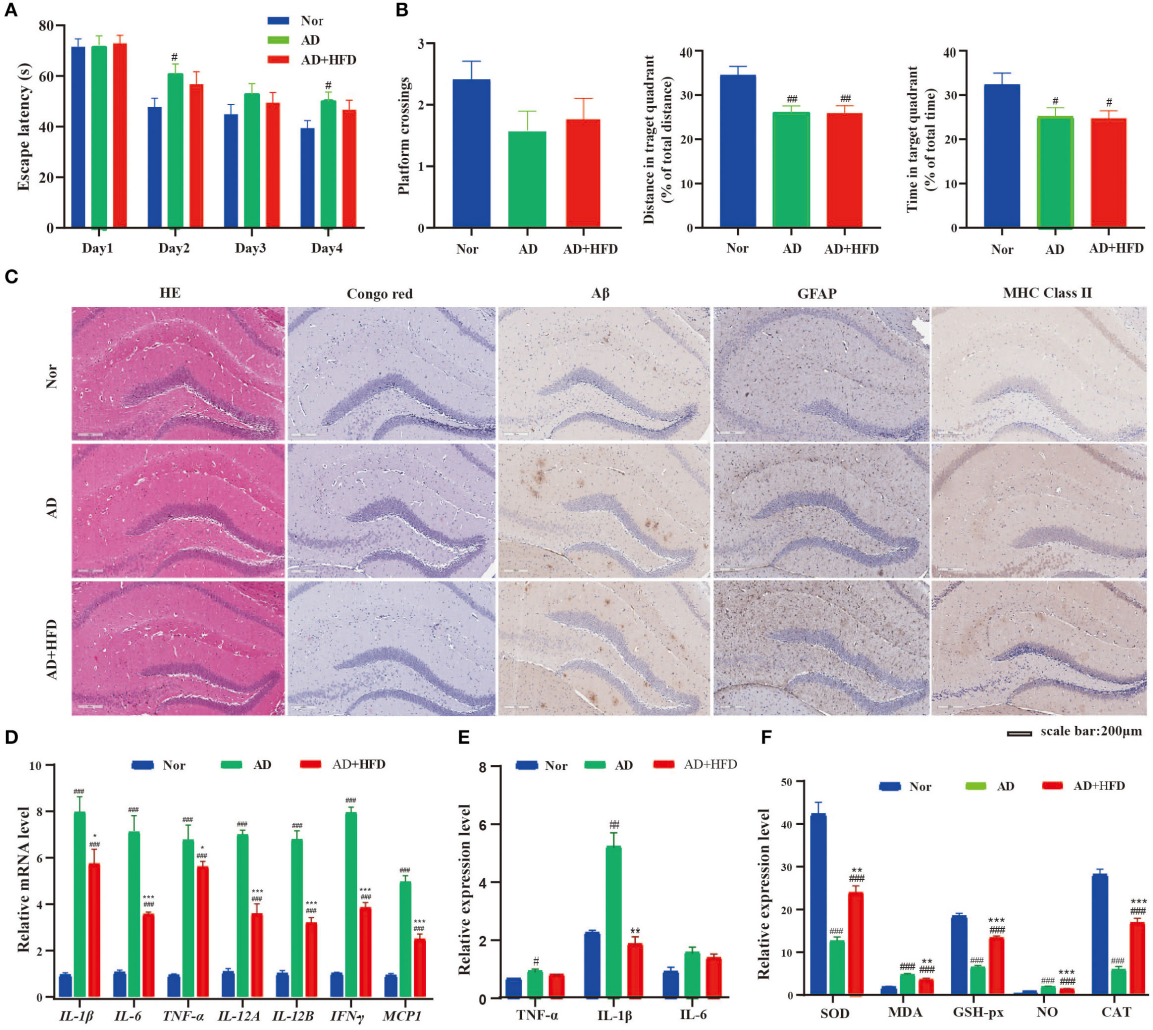
The effect of HFD on APP/PS1 mice cognitive impairment and cerebral pathology. **(A)** Escape latency during spatial acquisition training, **(B)** the platform crossing number, distance traveled percentage in the target quadrant and time spent percentage in the target quadrant in the spatial probe test. HFD had no effect on Aβ level, but HFD could relieve the inflammation in APP/PS1 mice. **(C)** The HE and Congo red stain in mice brain tissue, Aβ deposition was determined by Congo red stain and immunohistochemical technique. And the level of GFAP and MHC class II in brain tissue detected by immunohistochemical method. The expression of inflammatory cytokine detected by RT-PCR **(D)** and Elisa **(E)** method, respectively. And the indicators of oxidative stress **(F)** in brain tissue. ^#^ *p* < 0.05, ^*##*^ *p* < 0.01, ^*###*^ *p* < 0.001 (compared to Nor group); *p < 0.05, **p < 0.01, ***p < 0.001 (compared to AD group).

In the MWM test, the sequential changes in the average escape latency during spatial acquisition training are shown in Figure 1A. AD mice displayed markedly longer average escape latency compared to normal control mice on days 2 and 4, but AD + HFD mice were not significantly different. In the spatial probe test, the platform crossing number in AD and AD + HFD mice was lower compared to that in the control group. The percent distance and time spent in the target quadrant were significantly lower than those in the Nor group (Figure 1B). However, MWM test results in the AD + HFD group were similar to those in the AD group, indicating that there was impairment of spatial memory capacity in AD mice with no aggravation in response to treatment with a high-fat diet.

In HE staining of brain tissue, morphological lesions were not apparent in all groups. Next, we evaluated Aβ deposition in the brain by Congo red staining. The AD and AD + HFD groups exhibited obvious Aβ deposition. This result was in agreement with the immunohistochemistry in which the levels of Aβ were significantly increased in APP/PS1 mice compared to the Nor group (Figure 1C). Abnormal accumulation of Aβ induces excessive activation of microglia in the brain, which is associated with neuronal injury in AD. We next assessed whether a high-fat diet promotes inflammation in the brain. By immunohistochemistry staining, GFAP and major histocompatibility complex (MHC) class II expression were restricted to the hippocampal area in wild type mice but were overexpressed in the hippocampus and cortex of AD and AD + HFD mice. Both microglia and astrocytes present antigens via MHC class II molecular complexes with self-peptides to cause infiltration of CD4^+^ T cells in drainage lymphoid tissues that stimulate the adaptive immune response in the CNS (Das and Chinnathambi, 2019). Microglial cells are first primed by intracellular accumulation of Aβ, and prior to plaque deposition, classical markers of microglial activation, such as MHC class II, inducible nitric oxide synthase, and CD40, are already upregulated in the hippocampus of transgenic mice. The inducible, neuron-specific cyclooxygenase 2 (COX-2) enzyme is upregulated and specifically expressed by neurons in close relationship with Aβ-bearing cells (Ferretti et al., 2012).

#### A High-Fat Diet Relieves Inflammation and Oxidative Stress in Brain Tissue

To further assess the effect of high-fat diet on brain inflammation, we measured the mRNA expression of different proinflammatory cytokines, namely, *IL-1*β*, TNF-*α*, IL-6, IL-12A, IL-12B, IFN-*γ, and *MCP-1*, that were upregulated in the AD and AD + HFD groups. Surprisingly, we found that the mRNA levels of proinflammatory cytokines were significantly reduced in response to high-fat diet exposure (Figure 1D). Inflammatory cytokine levels were also detected by ELISA, and TNF-α and IL-1β were significantly increased in the AD group, while IL-1β levels were significantly reduced in the AD + HFD group (Figure 1E). Taken together, these results confirm that the effects of relieving inflammation might be associated with high-fat diet exposure.

Indicators of severe oxidative stress, that is, SOD, CAT, and GSH-px, exhibited significantly decreased expression in the AD and AD + HFD groups, and the levels of NO and MDA were strikingly elevated (Figure 1F). MDA is a marker for lipid peroxidation in the brain, and higher levels in APP/PS1 mice indicated disordered lipid metabolism. Compared to AD mice, the above indexes were significantly reversed in the AD + HFD group, suggesting that a high-fat diet may moderate oxidative stress to some extent.

#### High-Fat Diet Increases Body Weight, Serum Lipid Levels, and Inflammation

**Figure 2:**
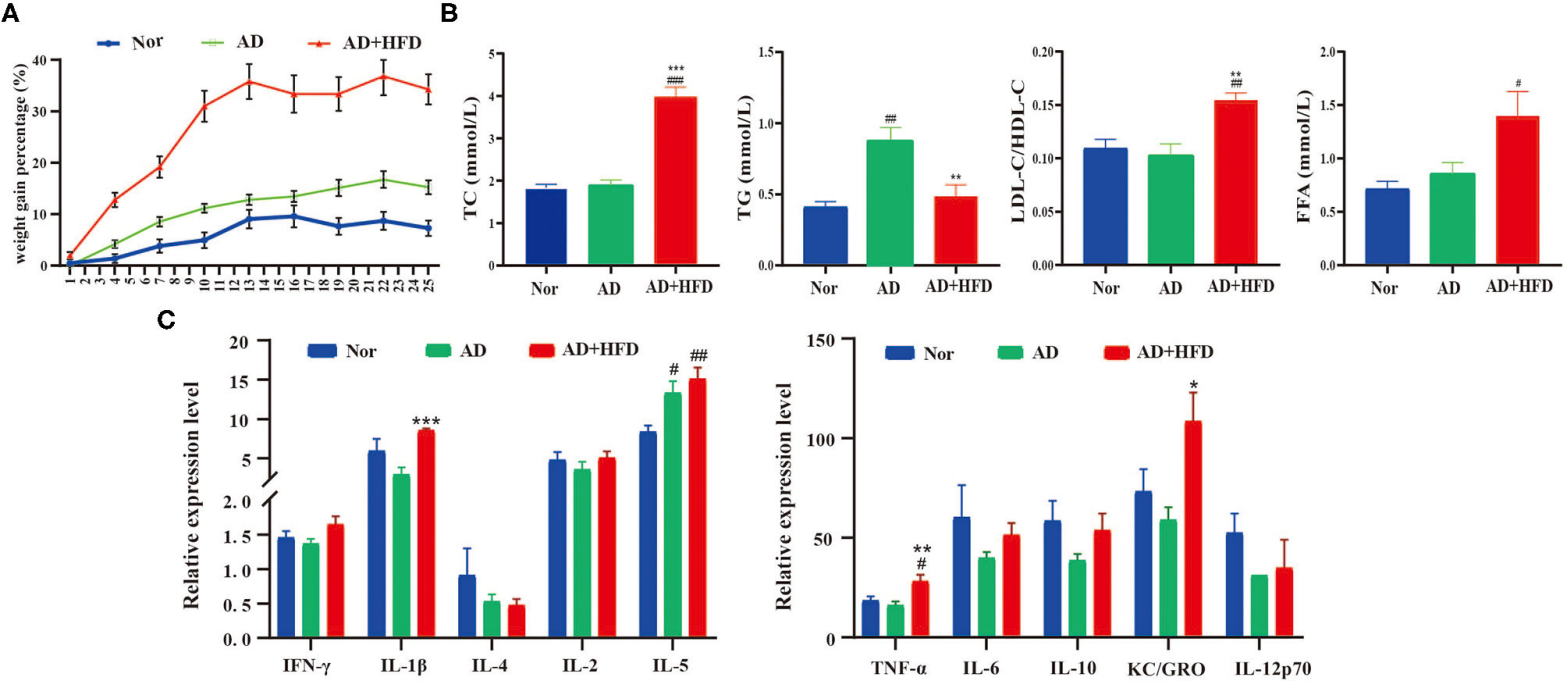
The effect of high fat diet on APP/PS1 mice body weights, serum lipid and inflammation level. **(A)** The weight gain percentage with either normal diet or HFD for 6 months. **(B)** The level of total cholesterol, triglyceride, ratio of LDL-C/HDL-C, and FFA in mice serum. **(C)** Serum inflammatory cytokines were detected by MSD cytokine assays. ^#^ *p* < 0.05, ^*##*^ *p* < 0.01, ^*###*^ *p* < 0.001 (compared to Nor group); *p < 0.05, **p < 0.01, ***p < 0.001 (compared to AD group).

We next investigated whether HFD treatment was sufficient to trigger obesity and hyperlipidemia in APP/PS1 mice. After 6 months of high-fat diet, there was obvious weight gain in AD + HFD mice at a rapid pace compared to mice on chow diet (Figure 2A). Serum TC, TG, the ratio of LDL-C/HDL-C and FFAs were also largely increased, indicating that high-fat diet induced hyperlipidemia in AD + HFD mice. The concentration of serum TG increased markedly, suggesting abnormal lipid metabolism in the AD group (Figure 2B). Because obesity was accompanied by peripheral inflammation, we next sought to study the impact of HFD on peripheral inflammatory tone. MSD cytokine assays provide a rapid and convenient method for measuring the level of protein targets. The results revealed that IL-5 was significantly increased in APP/PS1 mice, while TNF-α, IL-1β, and KC/GRO were markedly increased in the AD + HFD group compared to the AD group (Figure 2C). This finding indicates that there was inflammation in the serum of HFD mice.

### Alzheimer's-Associated Metabolic Alteration

#### High-Fat Diet Modulates Neurotransmitter and Amino Acid Metabolism in Brain Tissue

**Figure 3:**
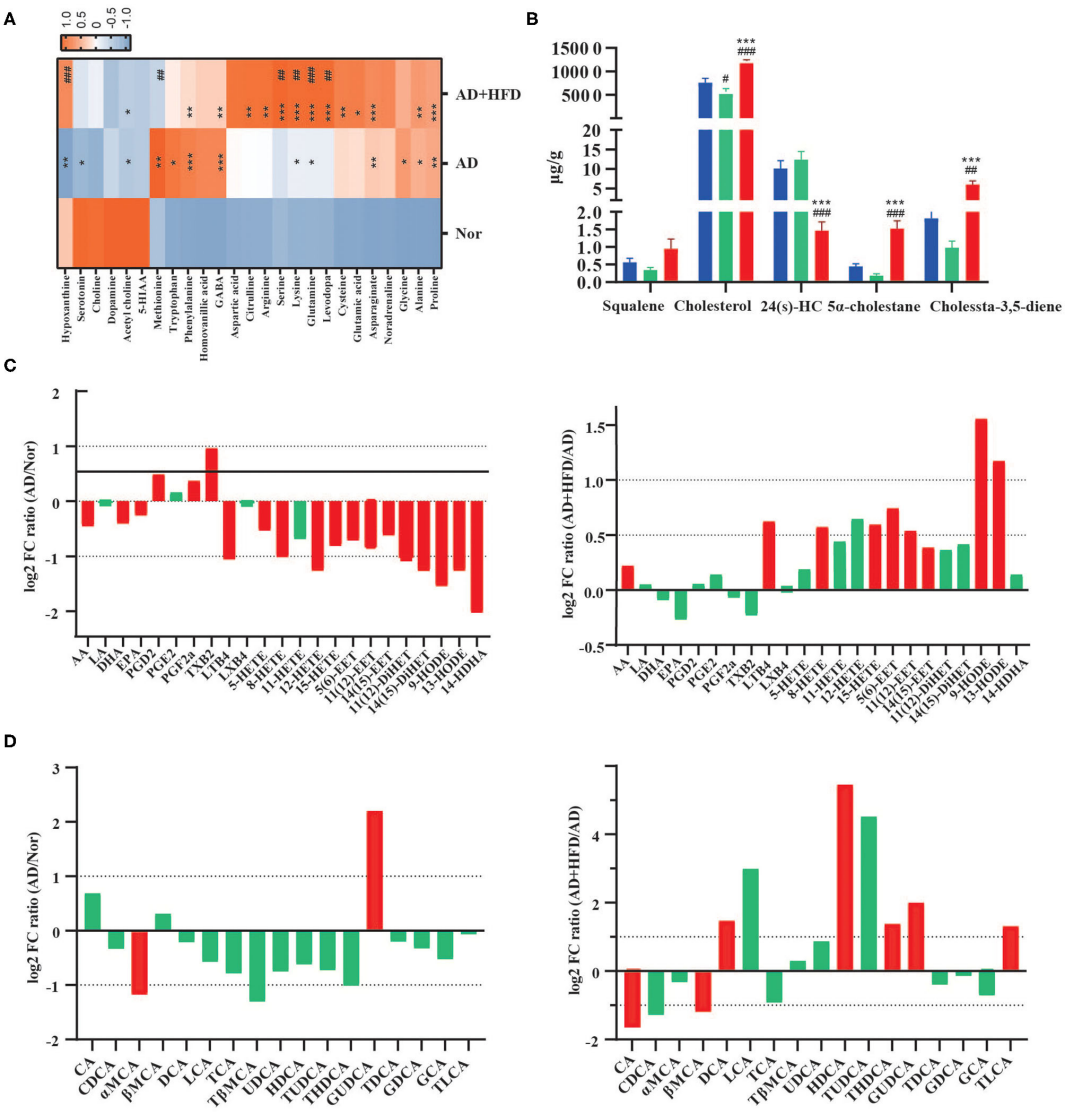
High fat diet altered the metabolite in AD mice. **(A)** The neurotransmitters and amino acids metabolism in brain tissue. *p < 0.05, **p < 0.01, ***p < 0.001 (compared to Nor group); ^#^ *p* < 0.05, ^*##*^ *p* < 0.01, ^*###*^ *p* < 0.001 (compared to AD group). **(B)** The level of cholesterol and its related compounds in brain tissue. ^#^ *p* < 0.05, ^*##*^ *p* < 0.01, ^*###*^ *p* < 0.001 (compared to Nor group); *p < 0.05, **p < 0.01, ***p < 0.001 (compared to AD group). **(C)** High fat diet-dependent changes in modulated polyunsaturated fatty acids and peroxilipidos metabolism in brain tissue samples (Log_2_ FC). **(D)** The concentration of 17 bile acids are dynamically changed in the serum (Log_2_ FC). The column in red had significant difference and the column in green had no significant difference. Data are presented as mean ± standard error of mean. ^#^compared to Nor group; *compared to AD group.

Amino acids have roles in neuronal signaling, energy production, and nitrogenous waste production and elimination. Aromatic amino acids in the brain function as precursors for the monoamine neurotransmitters serotonin and catecholamines (Fernstrom and Fernstrom, 2007). Brains and serum from patients with AD exhibit many alterations in amino acid levels and metabolism (Pan et al., 2016; Griffin and Bradshaw, 2017; Cui et al., 2020). Compared to the normal group, dopamine, acetyl choline, and serotonin were all decreased in APP/PS1 mice (Figure 3A), and acetyl choline was significantly different as well. GABA, glutamine, phenylalanine, lysine, asparaginate, proline, and alanine were significantly elevated in AD and AD + HFD mice. Additionally, serotonin and hypoxanthine were significantly decreased in AD mice, while glycine, tryptophan and methionine were significantly increased. Serine, levodopa, lysine, glutamine, and hypoxanthine were significantly increased in the HFD group compared to AD mice; however, methionine was significantly decreased.

All four principal nuclei of the monoaminergic systems undergo significant degeneration in AD (Trillo et al., 2013). In addition, bioanalysis of serotonin, dopamine and norepinephrine in the plasma after 8 weeks of HFD revealed that serotonin was significantly decreased in obese mice (Kim et al., 2013). In this experiment, we observed reductions in serotonin, 5-hydroxyindoleacetic acid and dopamine in APP/PS1 mice. The concentration of the sulfur-containing amino acid methionine was significantly decreased, and cysteine was significantly increased in AD + HFD mice compared to AD mice. Studies previously identified increased methionine levels in the CSF of MCI subjects (Kaddurah-Daouk et al., 2013), and dietary methionine supplementation resulted in increased Aβ and phosphorylated tau levels in the brain and cognitive impairment in wild type mice. In addition, cysteine supplementation caused a slight decrease in mTOR activity and inhibited the reduction in ROS in animals restricted by methionine (Gomez et al., 2015).

#### High-Fat Diet Increases Brain Cholesterol Levels

Cholesterols play a key role in the maintenance of brain health, and sufficient availability of cholesterol for synapse formation is a critical step in the structural and functional development of the nervous system (Hussain et al., 2019). Since peripheral and central cholesterol pools are not readily interchangeable, the brain has to produce cholesterol by *de novo* synthesis. In our research, cholesterol was significantly decreased in AD mice, but cholesterol and its precursor, squalene, were significantly increased in AD + HFD mice. Two reduction products of cholesterol, 5α-cholestane and cholesta-3,5-diene, were also present at higher levels in the AD + HFD group (Figure 3B). 24-Hydroxycholesterol (24S-HC) is the main mediator of cholesterol removal in the brain and represents classical feedback regulation of cholesterol homeostasis. 24S-HC serves as an activator of the nuclear transcription factors liver X receptors alpha and beta, which increase expression of cholesterol transport genes, including ABCA1 in both neurons and glia and ApoE in astrocytes (Martin et al., 2014). It serves as an intercellular signal that controls the delivery of astrocytic-derived cholesterol to neurons (Sun et al., 2016). Increased 24S-HC levels in both plasma and cerebrospinal fluid have been observed at early stages of AD, and as the disease progresses, 24S-HC levels decrease, possibly reflecting extensive neuronal loss and resultant loss of the CYP46A1 enzyme. Combined with the higher levels of squalene, lower levels of 24S-HC suggested that synthesis of cholesterol may be improved or that elimination may be suppressed by a high-fat diet.

#### High-Fat Diet Modulates Polyunsaturated Fatty Acid and Peroxilipid Metabolism

PUFA levels and the signaling pathways they regulate are altered in various neurological disorders, including AD and major depression diseases. PUFAs participate in signal transduction, either directly or after enzymatic conversion to a variety of bioactive derivatives (mediators) (Bazinet and Laye, 2014). In our research, arachidonic acid (AA), docosahexaenoic acid (DHA) and eicosapentaenoic acid (EPA) were significantly decreased in APP/PS1 mice. The mediators PGD2, PGE2, PGF2α, and TXB2, which are produced by COX and are highly proinflammatory, were elevated relative to the normal group. However, LTB4, HETEs, EETs, DiHETs, and 14-HDHA were significantly decreased. Of note, HFD regulated both the pro- and anti-inflammatory lipid mediators AA, LTB4, 5-HETE, 15-HETE, 5(6)-EET, 11(12)-EET, 14(15)-EET, 9s-HODE, and 13s-HODE, and these findings were significantly reversed in response to high-fat diet treatment (Figure 3C).

#### High-Fat Diet Affects Peripheral Bile Acid Levels in APP/PS1 Mice

BAs are the end products of cholesterol metabolism produced by human and gut microbiome cometabolism. A total of 17 bile acids were detected in mouse serum; among them, GDCA, GCA and TLCA were semiquantitatively detected due to their low levels in mouse serum. Compared to the normal group, GUDCA was significantly increased and α-MCA was significantly deceased in the AD group. CA and βMCA were decreased by HFD treatment; however, the levels of DCA, HDCA, THDCA, GUDCA, and TLCA were significantly increased. Compared to the normal group, α-MCA was significantly decreased in APP/PS1 mice. However, compared to chow-fed controls, DCA, HDCA, GUDCA, and TLCA were significantly increased by HFD treatment (Figure 3D).

Bile acids may be helpful to further diagnose AD in plasma along with a broader pattern of biomarkers (Marksteiner et al., 2018). It was previously reported that decreased serum levels of a liver-derived primary BA and increased levels of bacterially produced secondary BAs and their conjugated forms were associated with reduced cognition (MahmoudianDehkordi et al., 2019). The ratios of secondary to primary BAs (DCA:CA, LCA:CDCA) were associated with worse cognition, and these ratios were increased in AD + HFD mice in our study. However, HFD also improved functional BAs, such as UDCA, HDCA and their taurine-conjugated BAs, which are considered neuroprotective agents (Yanguas-Casás et al., 2014; Li C. X. et al., 2019).

#### High-Fat Diet Alters the Gut Microbiome in APP/PS1 Mice

**Figure 4:**
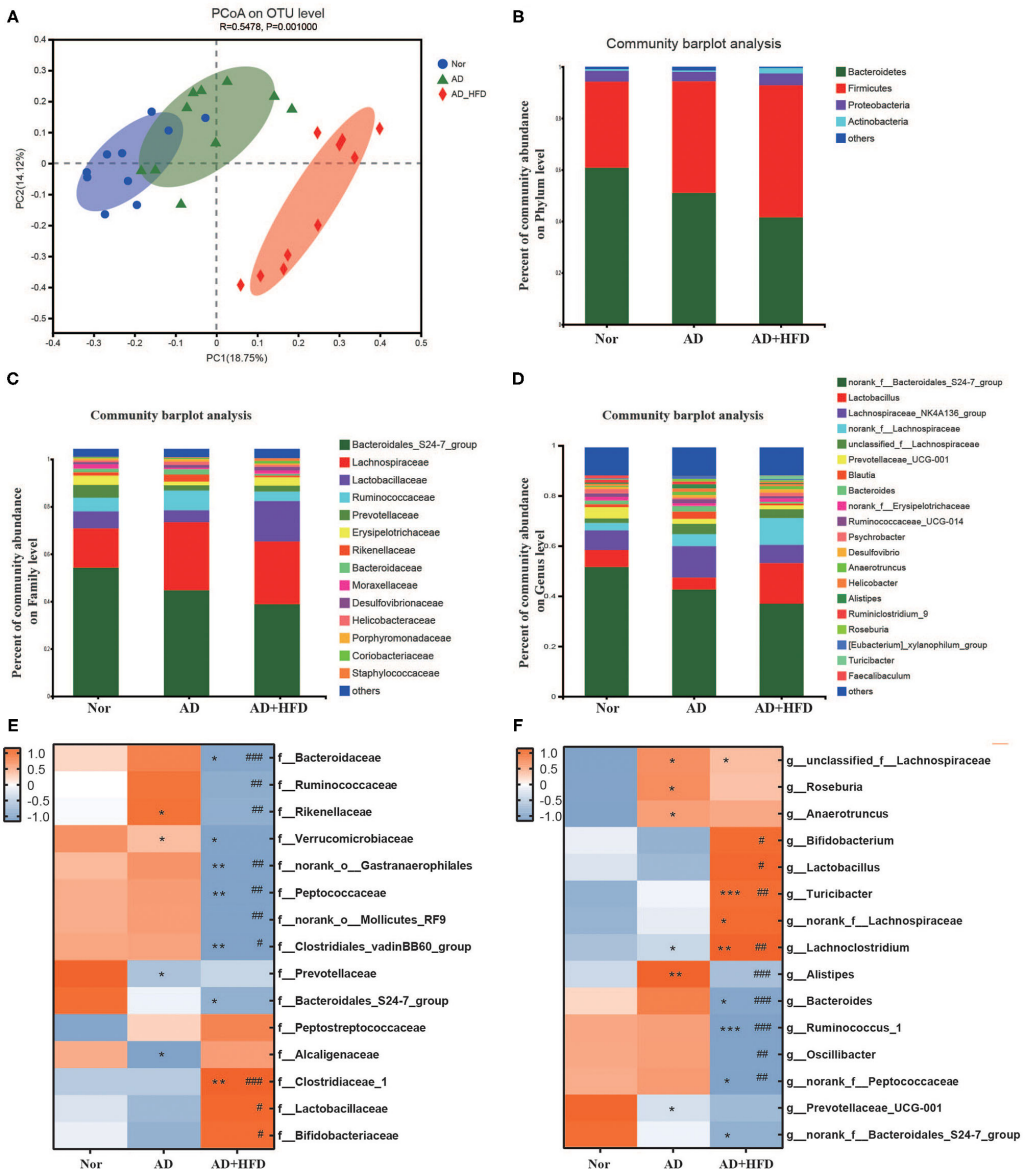
High fat diet altered the fecal microbiota in APP/PS1 mice. **(A)** PcoA based on the distance matrix of Bray-Curtis dissimilarity of the gut microbiota communities in the Nor, AD, and AD + HFD mice on the operational taxonomic unit (OTU) level. **(B–D)** The relative abundance of gut microbes of Nor, AD, and AD + HFD groups on Phylum, Family and Genus level, respectively. Heatmap of significant gut microbiota changes represented at the Family **(E)**, Genus **(F)** level. Colors on the heatmap indicate the relative abundance of gut microbiota. Gut microbiota with significant changes were chosen using a Wilcoxon rank-sum two-tailed test with *P* < 0.05. *p < 0.05, **p < 0.01, ***p < 0.001 (compared to Nor group); ^#^ *p* < 0.05, ^*##*^ *p* < 0.01, ^*###*^ *p* < 0.001 (compared to AD group).

Increased permeability of the gut and blood-brain barrier induced by microbiota dysbiosis may mediate or affect AD pathogenesis and other neurodegenerative disorders, especially those associated with aging (Jiang et al., 2017). In this study, the composition of the gut microbiome was characterized using traditional ecological measures, including OTUs, alpha diversity, and beta diversity. Taxon analysis revealed that the final OTU dataset for the Nor, AD and AD + HFD groups consisted of 895 OTUs classified into 172 genera, 75 families, 39 orders, 22 classes, and 12 phyla. For community richness estimates, we used the abundance-based coverage estimator (ACE) and Chao; these measurements use non-parametric modeling to calculate a conservative estimate of total OTU richness for each participant. The microbiome in mice fed a high-fat diet exhibited reduced richness, with ACE significantly decreased in the AD + HFD group compared to the control group. For community diversity metrics, we used the Simpson and Shannon indexes, and there was no significant decrease between the AD and AD + HFD groups (Supplementary Figure 1). With respect to beta diversity, Bray-Curtis-based principal coordinate analysis revealed distinct clustering of microbe communities among all three groups (Figure 4A). There was a difference between normal and AD mice, and remarkable changes in the microbiota community structure were induced by HFD intervention.

At the phylum level, AD and AD + HFD exhibited a decreased abundance of *Bacteroidetes* and increased abundance of *Firmicutes* compared to the normal group (Figure 4B). The Wilcoxon rank-sum test found that *Verrucomicrobia* was significantly decreased in both the AD and AD + HFD groups. In addition, *Tenericutes* and *Cyanobacteria* were also decreased in the AD + HFD group compared to the AD group. It was reported that Bacteroidetes and Verrucomicrobia are decreased in patients with AD, whereas Actinobacteria are slightly more abundant (Zhuang et al., 2018). Previous findings suggested that the production of neurotoxins by the phylum *Cyanobacteria* may play a role in the onset and development of cognitive dysfunctions (Mancuso and Santangelo, 2018). The present results indicated an alteration in the types of bacteria in both AD mice and HFD intervention mice. At the family level (Figure 4C), *Prevotellaceae* was significantly decreased and *Rikenellaceae* was significantly increased in AD mice. *Lactobacillaceae* was significantly increased and *Ruminococcaceae, Rikenellaceae*, and *Bacteroidaceae* were significantly decreased in the AD + HFD group compared to the AD group. Similarly, at the genus level (Figure 4D), *Prevotellaceae_UCG-001* was less abundant and *unclassified_f_Lachnospiraceae* and *Alistipes* were more abundant in AD mice. Compared to AD mice, *Lactobacillus* was increased, and *Bacteroides* and *Alistipes* were significantly decreased in AD + HFD mice. One study showed that Bacteroides and Alistipes were more abundant in patients with AD (Vogt et al., 2017). Notably, in our present research, HFD suppressed changes in Alistipes and *Bacteroides*.

Furthermore, the top 15 taxonomic genera that significantly differed in abundance among the Nor/AD/AD + HFD groups at the family and genus levels were analyzed (*P* < 0.05, Student's *t*-test). At the family level (Figure 4E), *Prevotellaceae* and *Bacteroidales_S24-7_group* were decreased in APP/PS1 mice, and *Peptostreptococcaceae* was enriched. Consistently, at the genus level (Figure 4F), *Prevotellaceae_UCG-001* and *norank_f_Bacteroidales_S24-7_* were decreased, whereas *unclassified_f_Lachnospiraceae, Roseburia*, and *Anaerotruncus* were increased in AD and AD + HFD mice. A high-fat diet alters the composition of the gut microbiota with a dramatic reduction in various bacterial groups, such as *Bacteroides, Ruminococcus_1, Oscillibacter*, and *norank_f_Peptococcaceae*. Increases in *Bifidobacterium, Lactobacillus, Turicibacter, norank_f_Lachnospiraceae*, and *Lachnoclostridium* were observed in AD + HFD mice compared to AD mice.

**Figure 5:**
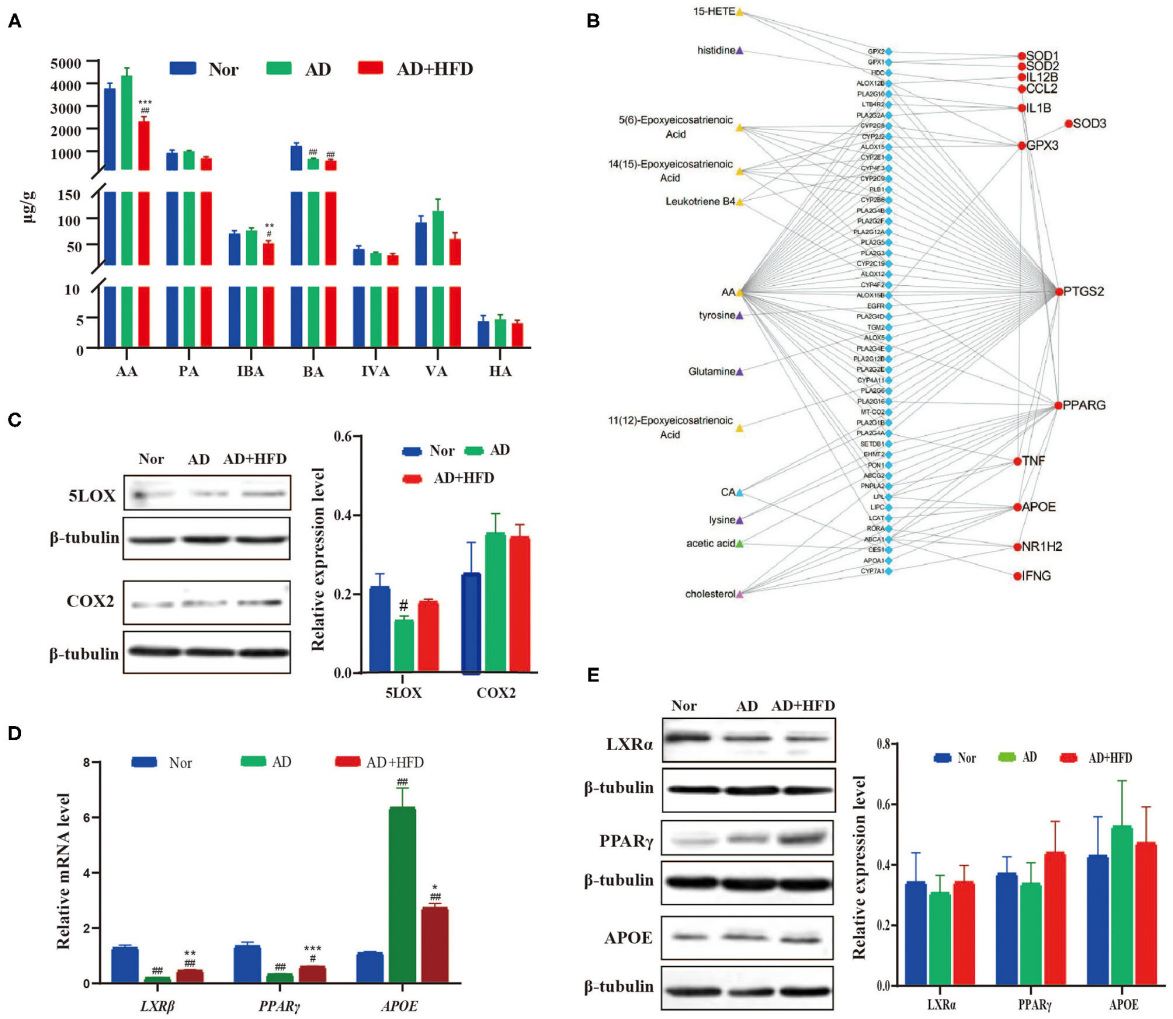
Network visualization analysis of metabolite-protein interaction in high fat treated mice. **(A)** The SCFA level changed by high fat diet. **(B)** Network visualization analysis of metabolite–enzyme (blue) –protein (red) interaction. The metabolites in which were significant changed in AD + HFD mice compared with AD mice. And the related protein detected by RT-PCR **(C)** and Western blot **(D,E)**.

The gut microbiota of Tg mice was highly dynamic, in great contrast to WT mice (Wang et al., 2019). In concert with the BA profile, the gut microbiota was partly restored by HFD. It was reported that *Oscillibacter* and *Alistipes* were negatively correlated with cognitive abilities, and *Lactobacillus* and *Bifidobacterium* were positively correlated with spatial learning and memory. In contrast, *Lactobacillus* and *Bifidobacterium* were negatively correlated, and *Oscillibacter, Alistipes*, and *Anaerotruncus* were positively correlated with proinflammatory factors (Wu et al., 2019; Xu et al., 2020). Therefore, these data demonstrated that HFD modulates neuroinflammation by altering microbiota abnormalities in APP/PS1 mice. Conversely, Akkermansia muciniphila treatment reversed high-fat diet-induced metabolic disorders, including fat mass gain, metabolic endotoxemia, adipose tissue inflammation, and insulin resistance (Everard et al., 2013), and our study revealed that *g_Akkermansia* was decreased in HFD mice. Furthermore, the main products of the fermentation of dietary fibers by anaerobic intestinal microbiota, SCFAs, were significantly decreased in response to high-fat diet (Wan et al., 2019). SCFAs can stimulate the NLRP6 inflammasome and ameliorate impairment of the intestinal epithelial barrier, resulting in protection against high-fructose diet-induced hippocampal neuroinflammation and neuronal loss (Li J. M. et al., 2019). Our research analyzed SCFAs in fecal samples and found that SCFAs were significantly decreased in APP/PS1 mice and that the total levels of SCFAs were decreased in response to high-fat diet treatment. AA and IBA were also markedly decreased by HFD intervention (Figure 5A).

### Network Visualization Analysis of Metabolite-Protein Interaction During HFD Intervention in AD

To explore the effect of altered metabolites during AD progression, we analyzed the correlation between metabolites and functional protein markers. Briefly, the differential metabolites in the AD and AD + HFD groups were searched in the HMDB database to identify their related enzymes. Then, the enzyme was connected with functional proteins using the STRING database. As a result, we found that AA and most of its mediators were related to COX-2 (also known as PTGS2), GSH-px (also known as GPX3) SOD, TNF and APOE. Cholesterol was related to PPARγ, APOE, LXRβ (also known as NR1H2), TNF, and IFNγ. There was also a potential correlation between CA and PPARγ, APOE, LOXβ, and acetic acid, as well as PPARγ and APOE (Figure 5B).

For further verification, brain tissues from 10-month-old APP/PS1 mice were collected and subjected to RT-PCR and western blot analyses. AA generated PGs and TXs through the COX-2 enzyme. LTs, LXs and HETEs are converted by lipoxygenase (LOX) enzymes. Western blot results showed that HFD reversed the elevation of COX-2 and 5-LOX levels in the brains of AD mice compared to the Nor group (Figure 5C). PGs and TXs, as well as COX-2, were increased, suggesting increased inflammation in APP/PS1 mice, and HFD relieved neuroinflammation in the brain. Next, we investigated whether the expression of APOE, LXR and PPARγ was changed in APP/PS1 mice. The results showed that *APOE* levels were increased in the AD and AD + HFD groups; however, *LXR*β and *PPAR*γ were significantly decreased. Compared to AD mice, *APOE* was significantly decreased in response to HFD treatment, and the expression of both *LXR*β and *PPAR*γ was increased (Figure 5D). Western blotting revealed that the variation in the protein levels was the same as that of the gene expression levels (Figure 5E). LXRs are transcription factors that control the expression of gene products involved in cholesterol homeostasis. Several direct LXR target genes are intimately linked to cholesterol transport, such as *ABCA1* and *APOE*. PPARγ induced cholesterol removal from macrophages through a transcriptional cascade mediated by the nuclear receptor LXRα (Chawla et al., 2001). In addition, reports have indicated that the regulation of the PPARγ-LXR-ApoE cascade may represent a significant molecular connection between adipocyte TG and cholesterol homeostasis (Yue and Mazzone, 2009).

### HLJDD Attenuates Pathology in HFD-Exposed AD Mice

**Figure 6:**
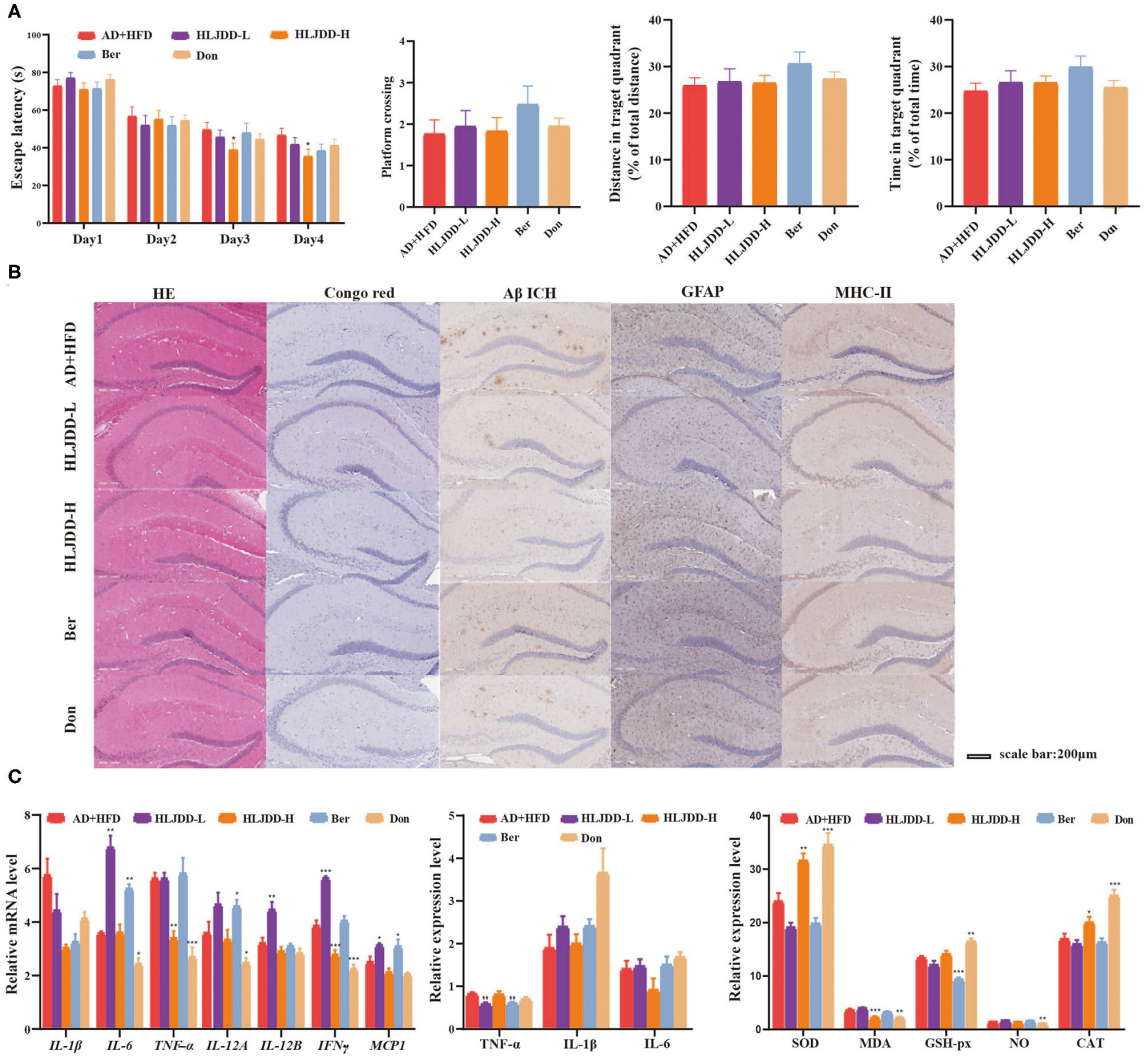
The effect of HLJDD on high fat diet treated APP/PS1 mice. Escape latency during spatial acquisition training **(A)**, the platform crossing number, distance traveled percentage in the target quadrant and time spent percentage in the target quadrant in the spatial probe test. **(B)** The HE and Congo red stain in mice brain tissue, Aβ deposition, the level of GFAP, and MHC class II in brain tissue detected by immunohistochemical method. **(C)** The expression of inflammatory cytokine detected by RT-PCR and Elisa method, respectively. And the indicators of oxidative stress in brain tissue. *p < 0.05, **p < 0.01, ***p < 0.001 (compared to AD+HFD group).

Our previous studies of the underlying therapeutic mechanism of HLJDD for the treatment of AD were associated with alleviating oxidative stress, inflammation, neurotransmitters, and energy metabolism (Gu et al., 2018, 2020; Wu et al., 2020). HLJDD also moderately improved cognitive competence in AD + HFD mice at a high dose (Figure 6A). In practical terms, the average escape latency was significantly decreased in the HLDD-treated group compared to the AD + HFD group on days 3 and 4 in the MWM. The percent distance, time spent in the target quadrant and number of platform crossings in the HLJDD group were increased, suggesting that cognitive ability was improved by HLJDD intervention. Aβ levels were markedly decreased in the hippocampus, illustrating that HLJDD may suppress Aβ deposition (Figure 6B). The expression of GFAP and MHC class II was reduced, and inflammation was reduced in brain tissue. Furthermore, *TNF*α and *INF*γ were significantly decreased in HLJDD-H mice. Moreover, indicators of oxidative stress were also moderately inhibited in high-dose HLJDD mice (Figure 6C). Additionally, weight gain was attenuated by drug intervention, and the serum lipid levels and cytokine IL-1β and KC/GRO levels in serum were lower compared to AD + HFD mice (Supplementary Figure 2).

HLJDD reversed the levels of lysine and glutamine, which were increased in the AD + HFD group, and HLJDD-L decreased the levels of levodopa (Supplementary Table 1). Cholesterol in brain tissue was also decreased by HLJDD (Supplementary Figure 3A). The high dose of HLJDD significantly decreased the levels of PGE2, PGF2a, and TXB2, indicating a neuroprotective effect (Supplementary Table 2). The expression of COX-2 and 5-LOX in the different groups had the same tendency as the downstream metabolites of PUFAs. However, the expression of LXR, PPAR and APOE was not significantly different (Supplementary Figure 3). In the periphery, HLJDD regulated BA and SCFA levels, as well as the gut microbiome. Specifically, CDCA, αMCA, TβMCA, TDCA, GDCA, GCA, TLCA, AA, and BA levels were reversed by HLJDD treatment (Supplementary Table 3, Supplementary Figure 2D). *Parasutterella, Alloprevotella, Alistipes, Rikenella*, and *Caproiciproducens* levels were also reversed in response to HLJDD (data not shown).

## Discussion

A high-fat/glucose diet was previously reported to induce biochemical changes related to increased AD biomarker burden and cognitive impairment (Lin et al., 2016; Kothari et al., 2017; Hill et al., 2019). However, other data have suggested no major effects of high-fat diet treatment on hippocampal neuroinflammation (Cremonini et al., 2019) or even a protective effect induced by HFD through mechanisms that involve improved BBB properties and brain morphology, as well as higher insulin receptor RNA expression and higher HDL cholesterol without a reduction in Aβ_1−42_ parenchymal load (Elhaik Goldman et al., 2018). To date, it is not possible to establish a causal relationship between diet and the development of AD with certainty because there are still many confounding factors and biases (David et al., 2014). More broadly, diet-related diseases will benefit from elucidating the links among nutritional, biliary and microbial dynamics (Grimm et al., 2013).

In the present study, cognitive competence and Aβ plaques were not aggravated in APP/PS1 mice by treatment with a high-fat diet. Endogenous substances, such as neurotransmitters and amino acid levels, were changed, and cholesterol and AA were increased in the CNS. Cholesterol affects Aβ generation, aggregation and subsequent neurotoxicity, as it is the primary component of lipid rafts in which the AD-associated proteins APP, β-secretase, and γ-secretase have been found (Geifman et al., 2017). While it is quite clear that pharmacological reduction of cholesterol levels in cells with normal cholesterol content inhibits amyloidogenesis in a variety of cell types and systems, it remains unclear whether conclusions from these studies can be extrapolated to the disease or normal aging contexts, especially taking into account the human studies showing that cholesterol levels are reduced in normal and pathological aging brains (Martin et al., 2014). The assumption about statins reducing the risk of AD (Zissimopoulos et al., 2017) is currently difficult to maintain because the reduction in AD risk varies across statin molecule status, sex, and race/ethnicity (Downs, 2018). Cholesterol levels in the serum/plasma and brain of patients with AD do not support cholesterol as a causative factor in AD (Salas et al., 2018). Furthermore, our study showed that microglia and astrocytes were activated and that the mRNA expression levels of inflammatory cytokines were inhibited in AD + HFD mouse brain tissue. Although originally identified for their role in cholesterol homeostasis, LXRs suppress inflammation through multiple direct and indirect mechanisms, some involving transactivation and others involving transrepression (Schulman, 2017; Fessler, 2018). Activation of PPARs reduces inflammation and confers neuroprotection, in part through their ability to minimize cell death and reduce mitochondrial dysfunction. PPAR activation may also enhance axonal growth and remyelination (Mandrekar-Colucci et al., 2013), as well as reduce Aβ levels in cell culture and AD animal models (Sastre et al., 2006). The pleiotropic effects of PPARγ activation are a promising efficacious candidate for neuropathologies (Villapol, 2018). Therefore, we cannot conclude that the high level of cholesterol in the AD + HFD group exacerbated AD symptoms.

Gut microbiota may affect brain function and behavior through the microbiota-gut-brain axis in a bidirectional interplay with both top-down and bottom-up regulation (Wang and Wang, 2016; De-Paula et al., 2018). Microbial involvement in the development of Aβ pathology (Harach et al., 2017) may contribute to neuroinflammation in AD (Giau et al., 2018). Alteration of the gut microbiota composition leads to the peripheral accumulation of phenylalanine and isoleucine, which stimulates the differentiation and proliferation of proinflammatory T helper 1 cells, which are associated with M1 microglia activation and contribute to AD-associated neuroinflammation (Wang et al., 2019). Host-microbe metabolic axes are thought to be involved in the regulation of multiple host metabolic pathways in which the levels of hormones, neurotransmitters, amines, SCFAs, lipid metabolites, and others are regulated by gut microbiome activity (Nicholson et al., 2012; Erny et al., 2017). As far as the HFD was concerned, alteration of the gut microbiota and subsequent changes in both the serum and brain BA profiles are mechanistically involved in the development of AD (Jia et al., 2020). An increased DCA:CA ratio in both the serum and brain was significantly associated with a worse cognition, and downstream effects might affect metabolic homeostasis and/or signaling functions in the human brain (MahmoudianDehkordi et al., 2019). In our study, the ratios of secondary to primary BAs, which reflected differences in gut microbiome enzymatic activity leading to altered production of secondary BAs, were increased in the AD and AD + HFD groups. Our study suggested that HFD might change CNS inflammation and metabolism partly by restoring the gut microbiota.

Bile acids and SCFAs have been reported to act as important regulators involved in the mechanisms by which gut microbiota affect lipid metabolism (Granado-Serrano et al., 2019). In our research, *Alistipes* was positively correlated with SCFAs and negatively related to DCA. *Oscillibacter* was positively related to IVA and negatively related to FFAs. Interestingly, *Clostridiales* was closely related to BAs, and most of the bacteria from *Ruminococcaceae* were negatively related to secondary bile acids, as the results of the correlation analysis show in Supplementary Figure 1B. However, the *Lachnospiraceae* family, such as *g_Lachnoclostridium, g_Marvinbryantia*, and *g_Lachnospiraceae_UCG-001*, is positively related to BAs. It was clear that microbial disturbances correlate with altered bile acid metabolism during the progression of various diseases and conditions (Joyce and Gahan, 2017). Other studies have shown that the gut microbiota also regulates the metabolism of bile acids that are ligands for the nuclear receptor farnesoid X receptor and the membrane receptor GPCR5, which are involved in the regulation of glucose, lipids, and energy balance (Pathak et al., 2018). In addition, our research showed that SCFAs were present at lower levels in AD + HFD mice. The depletion of butyrate-producing microbes by antibiotic treatment reduced epithelial signaling through the intracellular butyrate sensor PPARγ (Byndloss et al., 2017), which induced expression of genes involved in enterohepatic circulation and bile acid synthesis (Wang et al., 2018). As signaling molecules, SCFAs could adjust lipogenesis, gluconeogenesis and cholesterol synthesis notably through the G-protein coupled receptors GPR43/FFAR2 and GPR41/FFAR3 (Schoeler and Caesar, 2019). Dietary SCFA supplementation prevented and reversed high-fat diet-induced metabolic abnormalities in mice by decreasing PPARγ expression and activity (den Besten et al., 2015). As the main products of the fermentation of dietary fibers by anaerobic intestinal microbiota, SCFAs was a key note in the communication of gut and brain (Dalile et al., 2019).

## Conclusions

Our study showed that there were obvious cognitive impairment and Aβ plaques in APP/PS1 mice, regardless of their diet. However, neuroinflammation was slightly relieved by HFD treatment, which might have been mediated by increased levels of LXR/PPAR and altered gut microbes. It was further shown that a high dose of HLJDD exerted more effective anti-inflammatory and lipid-lowering effects, with potential effects on the pathophysiological cascade of Alzheimer's disease.

## Data Availability Statement

The data presented in the study are deposited in the NCBI SRA BioProject repository, accession number is PRJNA724708.

## Ethics Statement

The animal study was reviewed and approved by Beijing animal science Biotechnology Co., Ltd.

## Author Contributions

BB, BL, and HZ conceived the study concept and design and are guarantors of the article. HZ, XF, and JZ contributed to the data collection and elaboration, writing, and approval of the manuscript. XG, XW, HW, NS, and JY contributed to the methodology, discussion and writing and approval of manuscript. YY and FG contributed to the immunohistochemistry and network visualization analysis. YZ contributed to the valuable revision of the manuscript. The corresponding author attests that all listed authors meet authorship criteria and that no others meeting the criteria have been omitted.

## Conflict of Interest

The authors declare that the research was conducted in the absence of any commercial or financial relationships that could be construed as a potential conflict of interest.

## Abbreviations

AD: Alzheimer's disease
HFD: high-fat diet
HLJDD: Huanglian Jiedu decoction
Bas: bile acids
MWM: Morris water maze
SCFAs: short-chain fatty acids
Ber: berberine
Don: donepezil
24S-HC: 24-Hydroxycholesterol
AA: arachidonic acid
DHA: docosahexaenoic acid
EPA: eicosapentaenoic acid
OTUs: operational taxonomic units
ACE: abundance-based coverage estimator.
