# Supplementary material for: High-Fat Diet Alleviates Neuroinflammation and Metabolic Disorders of APP/PS1 Mice and the Intervention With Chinese Medicine
Source: Front Aging Neurosci. 2021 Jun 8;13:658376. doi: 10.3389/fnagi.2021.658376 (PMC8217439; doi:10.3389/fnagi.2021.658376)
Supplement: Supplementary file 1 [file Table_1.docx]

Supplementary Material

**1 Supplementary Figures**


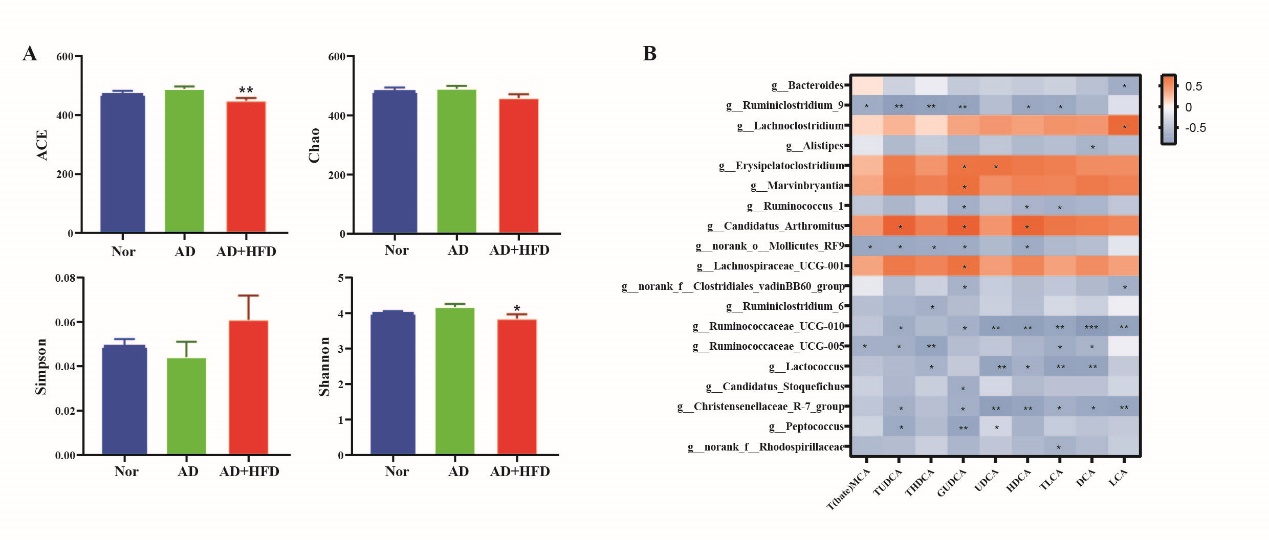


**Supplementary Fig.1** The alpha diversity of the gut microbiota (A) and the relation of BAs and microbiota (B). Heat map showing Pearson’ correlation coefficient with p <0.05 between several significant changed bile acids and genera bacteria. Red cells represent positive correlation and blue cells represent negative correlation.


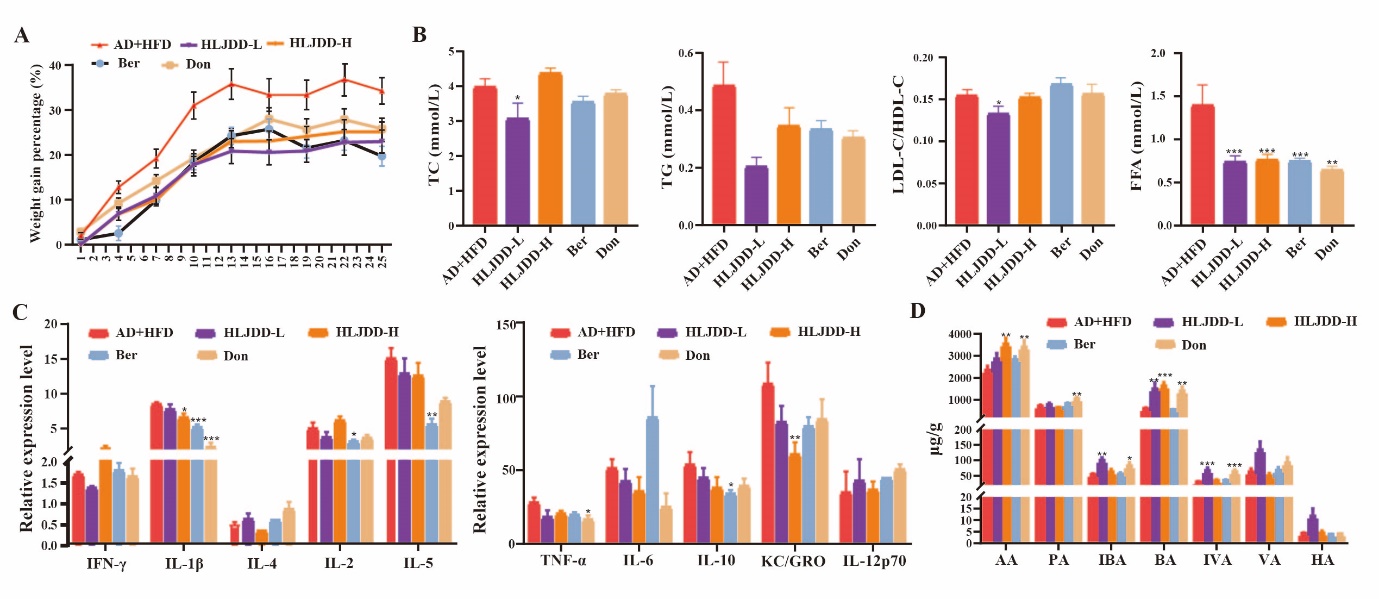


**Supplementary Fig.2** The effect of HLJDD on high fat diet treated APP/PS1 mice. (A) HLJDD effected on the weight gain percentage with HFD for 6 months. (B) The level of total cholesterol, triglyceride, ratio of LDL-C/HDL-C, FFA and inflammation cytokine (C) in mice serum. And the fecal SCFAs (D) changes by HLJDD treated. * p < 0.05, ** p < 0.01, *** p < 0.001 (compared to AD+HFD group).


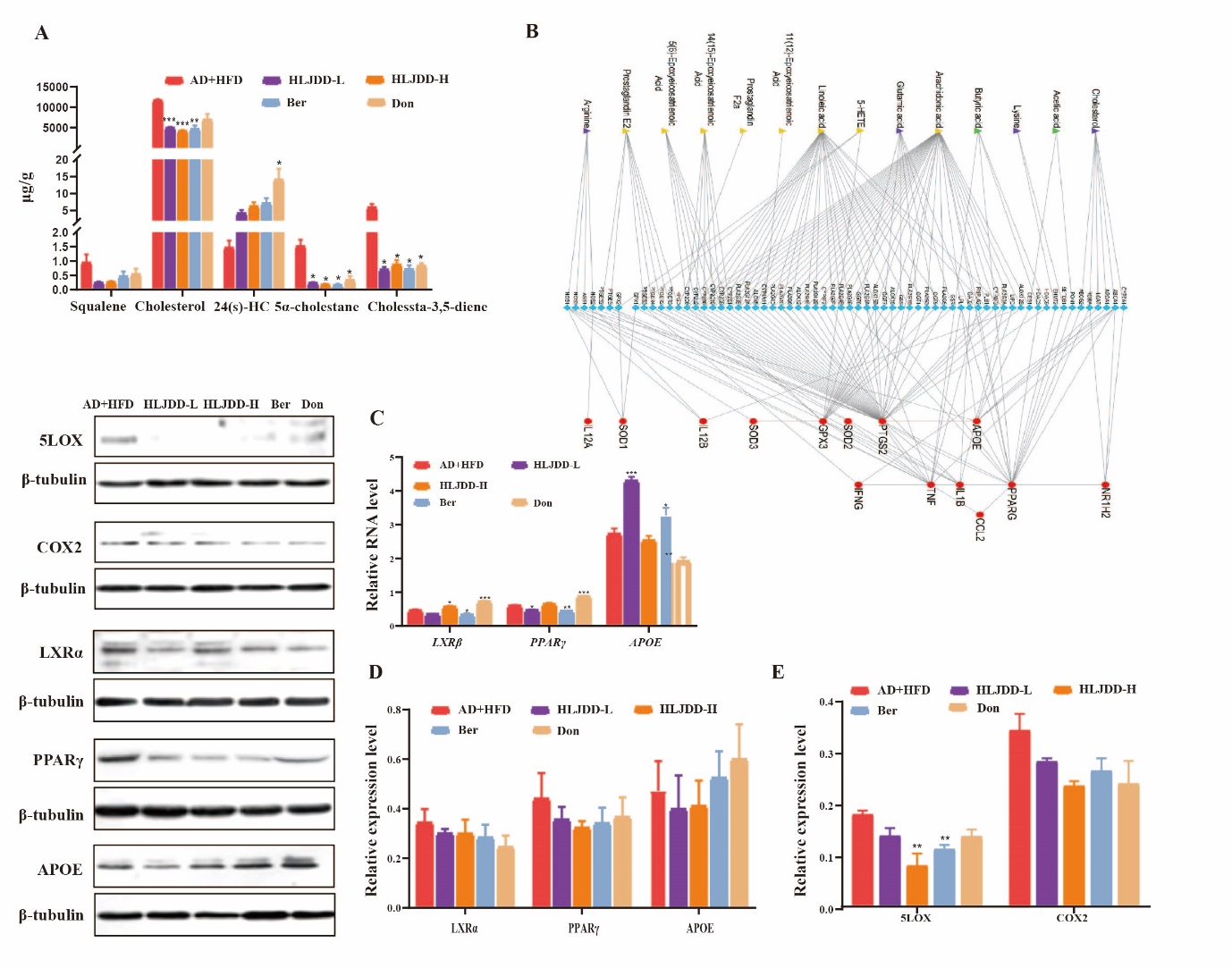


**Supplementary Fig.3** Network visualization analysis of metabolite-protein interaction in HLJDD treated mice. (A) HLJDD reversed the cholesterol and its related compounds in brain tissue by high fat diet. (B) Network visualization analysis of metabolite-enzyme (blue)-protein（red）interaction. The metabolites in which were significant changed in HLJDD-H mice compared with AD+HFD mice. And the related protein detected by RT-PCR (C) and Western blot (D, E). * p < 0.05, ** p < 0.01, *** p < 0.001 (compared to AD+HFD group).

**2 Supplementary tables**

Supplementary Table 1. Determination of neurotransmitter in different groups of mice brain tissue (μg/g)

|  | Compound | Normol | AD | AD+HFD | HLJDD-L | HLJDD-H | Berberine | Donepezil |
| --- | --- | --- | --- | --- | --- | --- | --- | --- |
| Absolute Quantification | 5-hydroxytryptamine | 0.94±0.22 | 0.68±0.20 | 0.75±0.17 | 0.78±0.24 | 0.93±0.15 | 0.91±0.35 | 1.02±0.28 |
|  | Cysteine | 5.41±1.24 | 6.45±0.83 | 7.10±1.55 | 7.65±1.82 | 6.15±1.99 | 5.94±1.19 | 6.48±0.90 |
|  | Alanine | 264.02±19.26 | 283.50±13.29 | 285.69±15.45 | 275.95±13.45 | 275.89±12.89 | 255.47±13.45 | 285.34±34.33 |
|  | Choline | 79.43±12.17 | 73.45±10.73 | 75.84±9.07 | 75.09±9.93 | 81.07±9.94 | 79.88±12.78 | 86.00±12.96 |
|  | Glycine | 38.45±7.06 | 49.61±6.83 | 48.53±7.04 | 46.43±10.54 | 47.19±5.63 | 39.04±7.72 | 47.47±14.55 |
|  | Homovanillic acid | 3.11±0.45 | 3.35±0.53 | 3.28±0.27 | 3.52±0.22 | 3.35±0.27 | 3.17±0.48 | 3.68±0.66 |
|  | Glutamic acid | 223.62±24.27 | 240.37±12.54 | 250.77±30.43 | 238.37±15.97 | 238.67±14.89 | 206.22±22.06 | 221.05±34.32 |
|  | Citrulline | 0.94±0.12 | 1.01±0.07 | 1.09±0.08 | 1.02±0.08 | 0.98±0.06 | 0.89±0.12 | 0.94±0.23 |
|  | Glutamine | 143.40±21.58 | 164.51±13.82 | 204.04±14.36 | 182.49±17.61 | 182.99±11.64 | 143.97±15.49 | 161.98±30.60 |
|  | Arginine | 18.75±2.80 | 20.45±1.91 | 22.45±1.72 | 20.11±2.23 | 19.58±1.41 | 16.77±2.27 | 18.50±4.97 |
|  | Lysine | 153.51±21.80 | 171.80±12.30 | 203.36±15.71 | 185.74±15.16 | 183.88±10.90 | 149.32±15.05 | 167.02±29.52 |
|  | Tryptophan | 4.13±0.62 | 4.59±0.69 | 4.38±0.29 | 4.45±0.35 | 4.05±0.42 | 4.20±0.53 | 3.98±0.44 |
|  | Aspartic acid | 157.12±16.17 | 165.52±20.71 | 174.09±16.83 | 168.97±14.82 | 154.76±9.48 | 136.22±16.19 | 161.92±44.69 |
|  | Asparaginate | 4.49±0.50 | 5.21±0.48 | 5.48±0.46 | 5.31±0.31 | 5.23±0.16 | 4.45±0.49 | 4.92±0.92 |
|  | Levodopa | 0.55±0.12 | 0.65±0.10 | 0.84±0.12 | 0.70±0.06 | 0.73±0.11 | 0.52±0.13 | 0.69±0.21 |
|  | Proline | 4.69±0.50 | 5.49±0.47 | 5.52±0.23 | 5.30±0.43 | 5.16±0.44 | 4.65±0.51 | 5.00±0.74 |
|  | Phenylalanine | 9.57±0.75 | 11.23±0.79 | 10.61±0.51 | 10.31±0.85 | 10.56±0.86 | 10.00±0.79 | 10.59±1.03 |
|  | Hypoxanthine | 0.55±0.12 | 0.42±0.07 | 0.60±0.09 | 0.44±0.07 | 0.52±0.10 | 0.45±0.12 | 0.49±0.12 |
| Relative Quantification | Dopamine | 2.42±2.01 | 1.75±0.37 | 1.65±0.37 | 1.64±0.66 | 1.74±0.63 | 2.02±1.01 | 2.17±1.64 |
|  | Methionine | 7.22±1.02 | 8.58±1.02 | 7.38±0.58 | 8.02±0.80 | 7.19±0.45 | 6.51±0.66 | 7.05±1.08 |
|  | Serine | 30.59±4.95 | 32.35±2.42 | 37.36±2.47 | 35.04±2.03 | 34.41±2.56 | 30.14±2.68 | 33.86±5.87 |
|  | γ-aminobutyric acid | 179.74±22.64 | 217.20±20.48 | 205.81±16.59 | 210.79±14.31 | 212.02±13.59 | 200.59±25.35 | 213.53±31.66 |
|  | Acetyl choline | 1.35±0.59 | 1.01±0.23 | 1.02±0.25 | 1.18±0.29 | 1.08±0.20 | 1.23±0.27 | 1.36±0.37 |
|  | Noradrenaline | 231.58±18.22 | 239.39±19.66 | 242.54±19.76 | 228.45±13.55 | 235.17±16.53 | 227.01±21.02 | 237.65±29.19 |
|  | 5-hydroxyindoleacetic acid | 0.52±0.05 | 0.50±0.07 | 0.50±0.08 | 0.54±0.07 | 0.46±0.06 | 0.57±0.10 | 0.58±0.15 |

Supplementary Table 2. Determination of PUFAs and their metabolites in different groups of mice brain tissue (ng/g)

| Compound | Normol | AD | AD+HFD | HLJDD-L | HLJDD-H | Berberine | Donepezil |
| --- | --- | --- | --- | --- | --- | --- | --- |
| AA | 108272.98±11649.88 | 79056.27±11200.26 | 92028.68±8010.05 | 90015.61±5319.44 | 87613.66±6026.27 | 103571.18±7841.12 | 90779.06±5941.34 |
| DHA | 47242.36±3944.24 | 35727.44±3205.17 | 33596.04±4422.16 | 31897.43±2401.39 | 31949.96±2428.85 | 43623.39±4869.75 | 36854.15±4586.56 |
| EPA | 2345.83±452.25 | 1960.54±330.02 | 1629.92±265.58 | 1230.36±153.79 | 1438.25±330.82 | 1528.80±448.76 | 2225.38±125.15 |
| LA | 61023.73±12028.21 | 58376.35±7133.15 | 60426.82±5634.05 | 54631.02±6551.20 | 51841.21±8234.75 | 53944.97±3225.58 | 58343.75±8525.03 |
| 5-HETE | 77.99±12.08 | 54.04±20.52 | 61.50±15.79 | 48.40±9.63 | 45.99±10.05 | 59.94±12.45 | 56.99±13.64 |
| 8-HETE | 35.48±7.12 | 17.59±5.76 | 26.12±5.21 | 19.77±3.86 | 24.35±6.21 | 22.40±6.07 | 24.90±5.61 |
| 11-HETE | 65.18±8.65 | 40.57±9.78 | 55.01±8.85 | 49.87±6.93 | 46.54±7.94 | 59.01±8.41 | 61.15±19.02 |
| 12-HETE | 137.54±43.63 | 57.50±21.35 | 89.86±21.78 | 78.47±6.87 | 78.26±17.26 | 154.45±43.01 | 150.53±31.38 |
| 15-HETE | 162.79±29.42 | 92.99±29.68 | 140.29±30.57 | 112.28±22.07 | 115.30±26.09 | 126.34±23.65 | 137.43±26.64 |
| 5(6)-EET | 10.24±2.03 | 6.27±1.65 | 10.48±3.05 | 10.72±2.60 | 13.78±2.46 | 5.12±1.04 | 6.20±1.47 |
| 11(12)-EET | 3.47±0.37 | 1.91±0.25 | 2.77±0.41 | 3.25±0.55 | 3.93±0.46 | 2.54±0.41 | 3.26±0.61 |
| 14(15)-EET | 4.41±0.49 | 2.88±0.39 | 3.76±0.46 | 4.72±0.57 | 5.48±0.57 | 3.45±0.35 | 4.58±0.68 |
| 11(12)-DiHET | 4.53±1.00 | 2.17±0.68 | 2.79±0.80 | 2.31±0.92 | 2.65±0.30 | 2.92±0.93 | 2.39±0.40 |
| 14(15)-DiHET | 4.70±0.97 | 1.96±0.65 | 2.61±0.72 | 3.11±1.02 | 2.74±0.75 | 2.61±0.70 | 2.64±0.74 |
| PGD_2_ | 87.50±15.73 | 122.36±27.03 | 127.02±19.48 | 125.72±26.53 | 118.13±10.28 | 109.73±27.83 | 138.63±34.73 |
| PGE_2_ | 77.32±12.72 | 86.37±21.38 | 95.16±17.30 | 94.06±16.55 | 78.64±4.77 | 93.07±15.35 | 96.65±17.14 |
| PGF_2α_ | 60.45±7.33 | 77.96±11.67 | 74.30±6.25 | 63.73±11.94 | 60.69±5.92 | 80.58±21.06 | 64.00±9.55 |
| LTB_4_ | 5.81±1.49 | 2.80±1.07 | 4.31±1.21 | 3.99±1.14 | 4.53±1.43 | 3.91±1.49 | 4.48±0.96 |
| TXB_2_ | 30.36±6.19 | 58.97±16.81 | 50.31±7.37 | 49.19±9.01 | 34.76±4.34 | 50.21±13.10 | 51.85±5.89 |
| LXB_4_ | 96.78±16.83 | 91.96±21.30 | 91.31±9.87 | 102.99±14.88 | 95.61±10.14 | 105.09±18.64 | 111.38±25.54 |
| 9-HODE | 88.33±31.41 | 30.42±11.25 | 89.35±32.04 | 73.86±18.92 | 72.25±34.14 | 46.60±14.81 | 70.45±24.64 |
| 13-HODE | 105.58±39.15 | 44.09±12.29 | 99.25±30.30 | 90.18±19.74 | 97.10±41.62 | 60.32±17.38 | 95.18±34.15 |
| 14-HDHA | 25.1±3.36 | 6.18±1.66 | 6.81±1.86 | 8.48±1.28 | 8.02±1.62 | 17.99±4.35 | 14.74±2.54 |

Supplementary Table 3. Determination of BAs in different groups of mice serum (ng/mL)

|  | Compound | Normol | AD | AD+HFD | HLJDD-L | HLJDD-H | Berberine | Donepezil |
| --- | --- | --- | --- | --- | --- | --- | --- | --- |
| Absolute Quantification | TβMCA | 173.571+28.84 | 70.294+6.75 | 86.207+4.93 | 100.235+11.97 | 173.107+36.69 | 58.565+6.77 | 71.891+11.40 |
|  | TCA | 49.231+13.52 | 28.623+6.30 | 15.155+5.36 | 13.166+1.02 | 22.252+8.24 | 15.801+2.97 | 18.928+3.90 |
|  | TUDCA | 5.206+1.42 | 3.152+0.98 | 72.171+24.57 | 41.340+5.38 | 73.400+19.23 | 49.109+7.88 | 63.726+8.24 |
|  | THDCA | 8.676+2.15 | 4.312+0.81 | 11.217+1.69 | 8.572+1.13 | 12.822+1.70 | 12.371+0.76 | 13.201+2,04 |
|  | TDCA | 8.556+2.04 | 7.448+1.21 | 5.680+0.86 | 8.006+0.84 | 9.855+21.01 | 7.582+1.85 | 10.200+1.74 |
|  | GUDCA | --- | 0.450+0.08 | 1.795+0.44 | 1.355+0，34 | 1.795+0.34 | 1.382 +0.14 | 1.256+0.06 |
|  | UDCA | 19.717+4.96 | 11.753+2.72 | 21.499+4.43 | 15.342+4.26 | 22.544+8.82 | 42.024+10.87 | 15.616+4.00 |
|  | HDCA | 2.762+1.14 | 1.799+0.48 | 78.671+111.33 | 71.378+17.40 | 123.223+23.10 | 79.045+18.17 | 84.378+7.71 |
|  | αMCA | 150.458+23.11 | 66.681+11.46 | 53.525+7.36 | 50.629+14.11 | 114.415+28.15 | 82.821+18.12 | 48.200+3.89 |
|  | βMCA | 449.761+55.95 | 558.166+93.05 | 245.024+27.80 | 302.997+86.55 | 318.529+82.16 | 481.702+137.21 | 268.737+45.46 |
|  | CA | 25.710+6.52 | 41.377+7.15 | 13.275+1.27 | 5.617+1.31 | 22.768+5.83 | 28.444+6.72 | 5.226+1.48 |
|  | DCA | 2.847+0.96 | 3.290+0，38 | 6.837+0,72 | 4.301+0.90 | 8.331+2,26 | 8.736+1.58 | 4.216+0.87 |
|  | CDCA | 36.263+9.81 | 28.859+5.35 | 11.897+0.92 | 24.344+3.70 | 49.531+8.39 | 13.939+2.24 | 23.203+3.57 |
|  | LCA | 1.326+0.31 | 0.894+0.54 | 7.101+4.61 | 3.028+0.58 | 2.083+0.35 | 2.002+1.11 | 2.214+0.55 |
| Relative Quantification | GDCA | 0.691+0.06 | 0.553+0.04 | 0.503+0.04 | 0.556+0.03 | 0.529+0.03 | 0.492+0.07 | 0.517+0.11 |
|  | GCA | 1.527+0.22 | 1.063+0.18 | 0.652+0.10 | 0.576+0.08 | 0.813+0.16 | 0.656+0.03 | 0.587+0.04 |
|  | TLCA | 0.392+0.06 | 0.395+0.08 | 0.980+0.15 | 0.646+0.09 | 0.840+0.14 | 0.664+0.07 | 0.769+0.11 |
